# Objective understanding of front of pack warning labels among Mexican children of public elementary schools. A randomized experiment

**DOI:** 10.1186/s12937-022-00791-z

**Published:** 2022-07-22

**Authors:** Alejandra Contreras-Manzano, Alejandra Jáuregui, Jorge Vargas-Meza, Claudia Nieto, Adriana Granich-Armenta, María de Lourdes Alemán Escobar, Armando G.-Olvera, Carlos Cruz-Casarrubias, Ana Munguía, Simón Barquera

**Affiliations:** Center for Research on Nutrition and Health, National Institute of Public Health, 62100 Cuernavaca, Mexico

**Keywords:** Front-of the-pack labeling, Warning labels, Children, Marketing, Cartoon characters, Objective understanding, Nutrition facts

## Abstract

**Background:**

Warning Labels (WL) highlight excessive amounts of critical nutrients in order to discourage consumption of unhealthful packaged food products. This study aimed to evaluate among Mexican school children, the objective understanding of traditional and numeric WL (aimed at small products) considered by the Mexican regulation, and whether cartoon characters influenced the understanding of WL. We also tested some communication strategies to facilitate the correct use of the WL.

**Methods:**

We carried out a randomized experiment in July 2019 in public elementary schools from Morelos, Mexico. Participants aged 6–13 years, were randomly assigned to one of four groups: 1) Nutrient Facts Panel (NF) (*n* = 120), 2) Nutrient Facts Panel with cartoon characters (NF + C) (*n* = 83), considered the control groups, 3) Warning Labels (WL) (*n* = 109), and 4) Warning Labels with cartoon characters (WL + C) (*n* = 96). After allocation, children assigned to both WL groups (WL or WL + C), were randomly required to watch two posters simultaneously or a video explaining how to correctly interpret WLs. Logistic regression models adjusted by sex, age and cluster (school) were fitted.

**Results:**

The percentage of children correctly choosing the healthiest or the unhealthiest option was higher for WL groups (56.8, 95%CI; 40.8–72.8) compared to NF groups (24.3, 95%CI; 20.4–28.3, *p* < 0.05). The understanding of traditional WL was higher (28.7, 95%CI: 22.8–35.4) than the numeric WL (19, 95%CI: 14.2–25.0, *p* < 0.05). But, correct answers for identifying healthy and unhealthy products were higher for numeric WL than for NF groups. Cartoon characters reduced the percentage of correct answers for choosing unhealthiest products (WL + C: 48.9, 95%CI: 25.6–72.4 vs WL: 58.7, 95%CI: 36.4–81.1, *p* < 0.05). The video was 2.23 times more helpful than the posters to the correct interpretation of the WL (*p* < 0.05).

**Conclusions:**

In scholar Mexican children, traditional and numeric WL were useful to identify healthier and unhealthier packaged products in comparison to NF, suggesting that both WL formats may effectively communicate the excessive content of nutrients of concern among children. Cartoon characters may reduce the objective understanding of the WL, underscoring the need to regulate advertising directed to children along with the implementation of front-of-pack labeling.

**Supplementary Information:**

The online version contains supplementary material available at 10.1186/s12937-022-00791-z.

## Introduction

Mexico is one of the leading countries worldwide in the obesity epidemic and has the highest increase rates in childhood obesity [1–3]. In total, 35.6 and 38.4% of Mexican children and adolescents, respectively, live with obesity [4]. Ultra-processed products, with high quantities of saturated fat and/or added sugar, have contributed to the development of this public health problem [5–7]. Among Mexican school-aged children and adolescents, ultra-processed products represent 34.3% and 35.5%, respectively, of the total calories consumed [8].

Warning Labels (WL) are a new labelling approach, highlighting excessive amounts of added sugar, sodium or saturated fats in processed foods or beverages in order to discourage the consumption of unhealthful products [9]. WL have been implemented in Chile (2016) [10], Peru (2018) [11] Mexico [12], Uruguay (2021) [13], and Argentina [14]. After 1 year of implementation in Chile (2017), purchases of “high-in” beverages decreased 22.8 mL/capita/day, whereas purchases of products without labels increased [15]. Some of these regulations also considered restrictions for marketing strategies and advertising to children for products with one or more WL [12, 16].

However, one of the limitations of the WL regulations implemented in Chile and Peru is that small products (<50cm^2^ in Peru and < 30 cm^2^ in Chile) are not labelled because WL do not fit in these small packages. Taking advantage of this exception, manufacturers in Peru reduced the size of some of their products to avoid WL [17, 18]. Therefore, many small products high in sugar, fat and/or calories (e.g., candies, chocolates and cookies), are not labelled with WL and, thus, may use cartoon characters or other marketing strategies directed to children, making this population more vulnerable to make unhealthy food choices [19]. Studies suggest that marketing strategies targeting children and youth may change perceptions of product taste, attract more attention [20], increase product’s appeal [21] and influence children’s food preferences, consumption patterns, and purchase requests. Further, cartoon characters help children recognize the brand [22, 23], and aim to create a positive attitude and loyalty towards the product [24]. In addition, food and beverages most frequently promoted to children are high in saturated fat, sugar or sodium [23].

WL are one of the most understood front-of-the-package labelling by adults across countries [25]. Evidence also indicates that WL are well understood among adolescents [26]. However, few studies have explored the understanding of WL among school-aged children. In Uruguay, school-aged children were more able to correctly identify a product with high content of a critical nutrient when using WL compared to GDA or Multiple Traffic Light (MTL) [27]. In Brazilian children the perceived healthfulness of an unhealthy product (i.e. chocolate flavoured milk and yogurt) decreased when using WL compared to the GDA system [28]. Additionally, scarce evidence exists regarding the influence of marketing strategies to children, such as cartoon characters, on WL understanding [27].

**Fig. 1:**
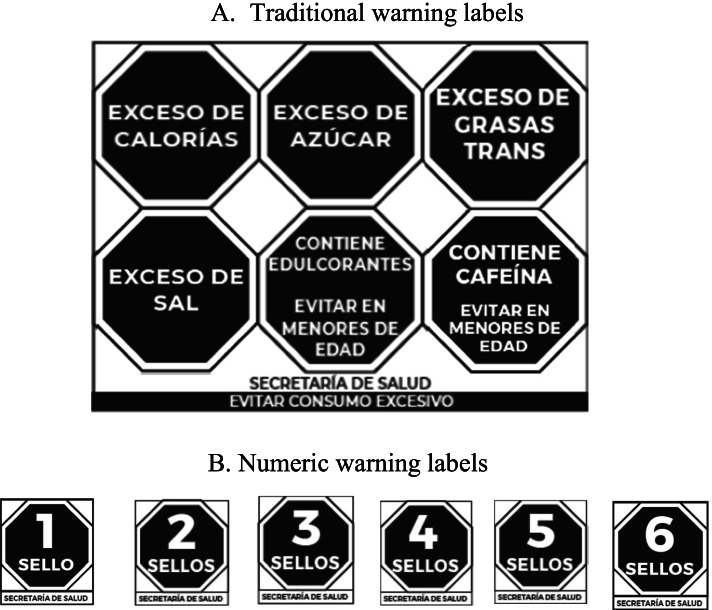
Warning labels as originally considered by the Mexican regulation, before it was reviewed and debated from August 2019 to January 24, 2020, when the modification was approved.** A** Traditional warning labels. **B** Numeric warning labels

Based on previous experiences and to prevent small products waving WL, the WL regulation in Mexico considers the implementation of traditional WL (‘Excess’ labels or labels in captions) for regular size pre-packaged products with excessive contents of added sugar, sodium or fat, as well as numeric WL for small products (< 40 cm^2^) or products in returnable packaging Fig. 1A  [12]. Numeric WL indicate the total number of WL a small product has with a single label (Fig. 1B). Based on the Mexican system, a similar system was recently implemented in Argentina [14]. However, to date no study has explored the understanding of numeric WL among consumers.

Communication strategies aiming to improve the use and understanding of front-of-pack nutritional labels are key aspects to be considered during the implementation of new regulations [29]. For example, in Chile a communication campaign was launched along with the implementation of WL. Studies indicate that over 90% of consumers understood WL at 6 and 18 months after their implementation [30] A randomized experiment in Peruvian children explored the use of an emoji to rate the emotions associated with a product, finding that WL had a greater effect than MTL in associating negative emoji for unhealthy products [31]. In Mexico, no studies have been developed to investigate the effectiveness of food labelling communication strategies among children.

We carried out a randomized experiment to investigate the objective understanding of traditional and numeric WL among Mexican school children, and whether cartoon characters influenced the understanding of WL. We also tested some communication strategies to facilitate the correct use of the WL.

## Material and methods

### Design

We carried out an unblinded randomized experiment with a split-plot design of four factors in July 2019 (Supplementary Fig. 1), before the new regulation for WL in Mexico was approved. The Ethics, Research and Biosafety committees of the Mexican National Institute of Public Health evaluated and approved this study (approval number: 7-68S4-P62–19).

### Recruitment

The study was conducted in three public elementary schools out of four schools selected by convenience in the state of Morelos, Mexico. Schools were invited to participate in the study after explaining to the school principals the study objectives, activities, benefits and potential risks for the children. Children from 6 to 13 years of age from all grades (i.e., from first to sixth grade in the Mexican school system) were invited to participate in the study. Written consent forms were sent home with children and only those with consent to participate by their parents or guardians were included. Before starting study procedures, children were asked to assent to participate in the study.

### Participant’s allocation

Participants (*n* = 410) were randomly assigned to one of four groups: 1) Nutrient Facts Panel (NF) (*n* = 120), 2) Nutrient Facts Panel with cartoon characters (NF + C) (*n* = 83), considered the control groups, or 3) Warning Labels (WL) (*n* = 109), and 4) Warning Labels with cartoon characters (WL + C) (*n* = 96), considered experimental conditions (Supplementary Fig. 1). The format of the WL considered in this study corresponded to the one originally proposed in the regulation, before it was reviewed and debated from August 2019 to January 24, 2020, when the modification was approved [12]. The original regulation considered 6 WL and numeric WL for products with a front-of-pack area of <10cm^2^ instead of the 5 WL and numeric WL for products with a front-of-pack area of <40cm^2^ outlined in the new regulation. Randomization was done with raffle tickets indicating children’s allocation (i.e., children randomly chose a ticket from a basket). Blinding of participants or researchers was not possible given the nature of the intervention. A group of five researchers and five fieldworkers carried out the study; school teachers were present during the activities with the participants.

#### Experiment

**Fig. 2:**
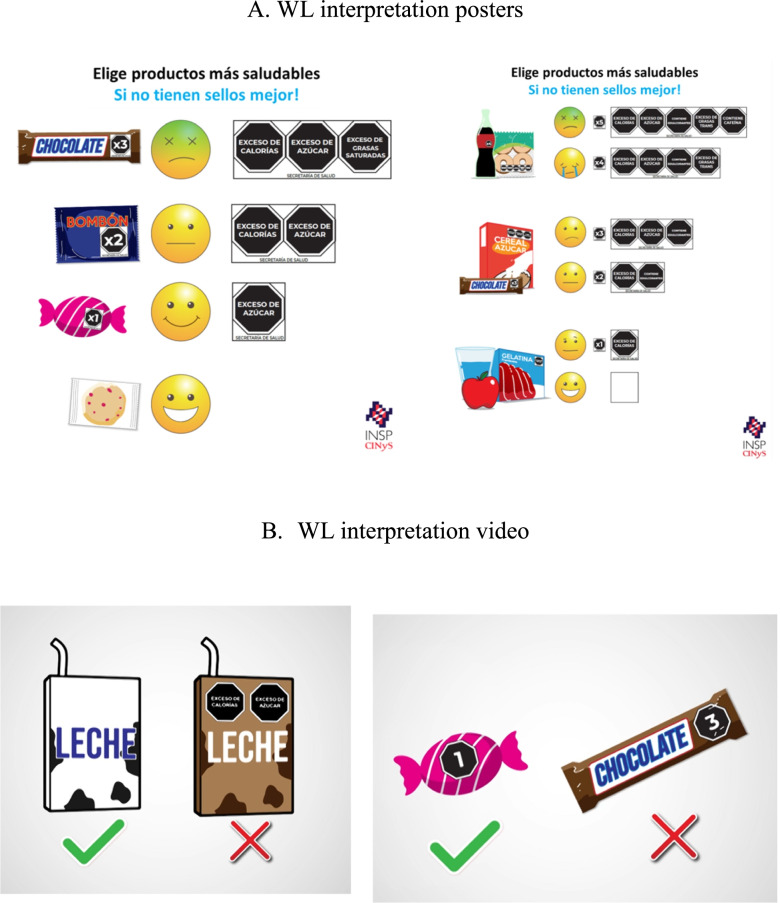
**A**. WL interpretation posters. **B** WL interpretation video

After allocation, children assigned to both WL groups (WL or WL + C) were randomly required to watch two posters, both displayed together on a desk, (Fig. 2A) or a video (Fig. 2B) explaining how to correctly interpret WLs (Supplementary Fig. 1). Children were not given any other explanation on how to interpret WL. Children allocated to other study groups (NF and NF + C) were not provided with any interpreting aids.

The video (Supplementary Video 1) showed examples of products with and without WL (Fig. 2B), visually displaying approval (i.e., a tick) or disapproval (i.e., a cross), respectively, and suggested choosing the healthiest food product (i.e., the one with no WL, with the fewest labels or the smaller number). It also mentioned that minimally processed foods were the healthiest [32]. On the other hand, the posters (50 × 60 cm) (Fig. 2A) displayed products along with smiling emoji if the product had no WL or dislike emoji if the product had two or more WL, based on previous emoji association with perceptions of healthiness with front of package labelling in children [31]. The key messages of both interpreting aids (i.e., video and posters) -“choose the healthiest” and “it is better if it does not have warning labels”-, were selected based on the Chilean government WL campaign [11, 29]. These messages were piloted and tested in school-age children before preparing the interpreting aids.

**Fig. 3:**
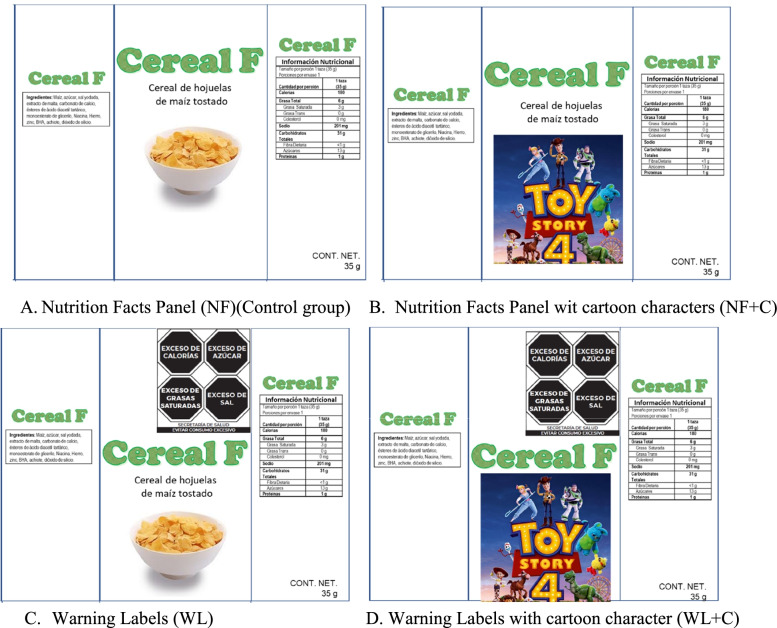
Example of a dummy product (breakfast cereal) with a front-of-pack area >10 cm^2^ by group of study. **A **Nutrition Facts Panel (NF). **B** Nutrition Facts Panel with cartoon characters (NF + C). **C** Warning Labels (WL). **D** Warning Labels with cartoon character (WL + C)

**Fig. 4:**
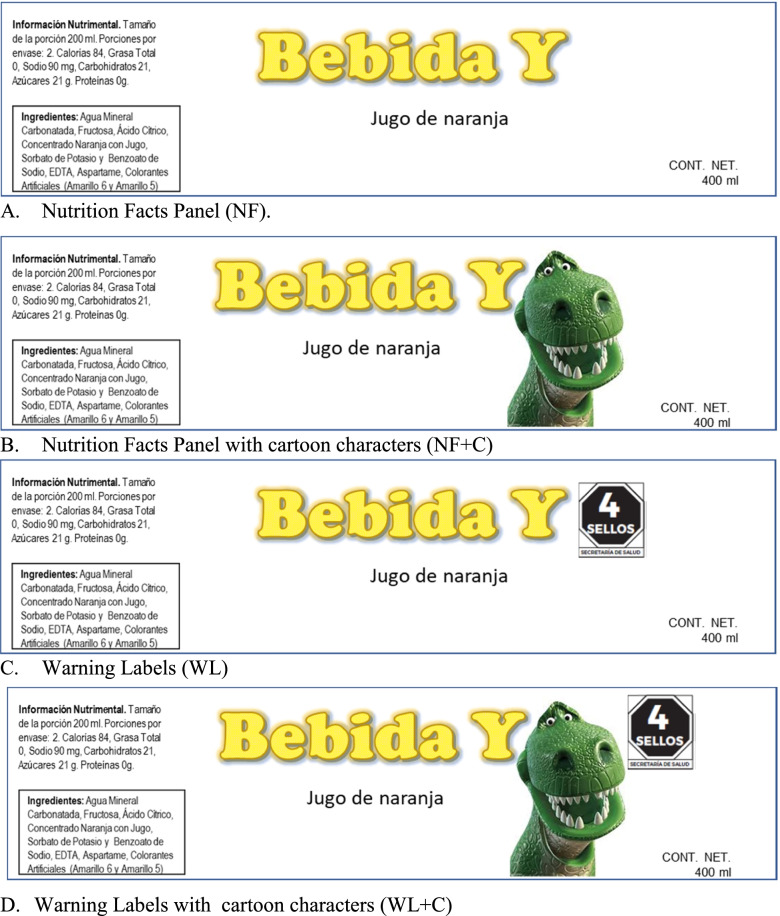
Example of a dummy product (orange juice) with a front-of-pack area <10 cm^2^ by group of study. **A** Nutrition Facts Panel (NF). **B** Nutrition Facts Panel with cartoon characters (NF + C). **C** Warning Labels (WL). **D** Warning Labels with cartoon characters (WL + C)

A set of fictitious products were used per study group. Each set contained a total of 42 food products from 6 food groups, with different packaging sizes: 3 regular size groups (beverages in disposable packaging, breakfast cereals, and dairy beverages) (Fig. 3A-D), and 3 small size products or products with returnable packaging groups (i.e., candies, cookies and beverages in returnable packaging) (Fig. 4A-D).

All packaging looked the same across study groups, except for the WL and/or the cartoon characters displayed. Products showed no brands, no other labels, claims or advertising. In order to include a range of products with varying nutritional quality, the nutritional information and ingredients list of similar real products was used. This information was used to evaluate the nutritional quality of food products, according to the Mexican Nutrient Profile (Supplementary Table 1). Additionally, the following variations were considered in the set of products used in each study group:

1. Warning labels: Products used in the NF group only displayed the Nutrient Facts Panel, while for WL groups (WL and WL + C) the products displayed the Nutrient Facts Panel and the corresponding WL, going from zero warnings (the healthiest product) to six warnings (the unhealthiest product). Traditional WL (Fig. 1A) were displayed in regular size food products, whereas numeric WL (Fig. 1B) were displayed in small size products or products with returnable packaging. For regular size food products (> 10 cm^2^) the Nutrient Facts Table in the back of the package was displayed (Fig. 3A-D).
2. Cartoon characters: Products used in cartoon characters’ groups (NF + C and WL + C) included one product with a cartoon character (Fig. 3B and D). This product displayed 4 WL in the WL + C group or the equivalent product in the NF + C. The candies group did not display neither the nutrition facts table nor the cartoon character in the product package.

Children were asked to sit in front of a table. Initially, children were shown a set of seven products corresponding to the first food group (e.g., breakfast cereals). For this purpose, children were required to close their eyes while products were set on the table. Then they were allowed to open their eyes and were asked: Which food product is the healthiest? Children chose one food product among the seven options. Then, children were asked: Which food product is the least healthy? Again, children chose one food product among the six remaining options. In both occasions, the time required to make a decision (i.e., time in seconds, starting when the child opened his/her eyes, up to when a decision was made) as well as the product chosen by the child were registered. This process was repeated for each of the remaining five food groups.

The order in which food groups were presented was randomly assigned using a total of six possible combinations for the three regular size groups and for the three small size groups. Additionally, the way in which the set of seven products was displayed on the table varied in order to avoid a sequence or order bias. Once their participation was concluded, children were rewarded with stickers and the fruit of their choice (e.g., a banana, apple or an orange). Additionally, the school received one soccer or volleyball ball per classroom as a retribution for participating in the project.

##### Outcomes

The percentage of children correctly identifying the healthiest product (i.e., product with no WL), the least healthy product (i.e., product with six WL) and both products (i.e., healthiest and least healthy product) was considered as the primary outcome. The time required to make a decision among study groups was considered as the secondary outcome.

### Covariates

Before allocation, children answered a brief questionnaire that collected socio-demographic characteristics like sex, age, literacy, and habitual food shopping location.

### Analysis

Based on previous studies among Mexican consumers [25, 26, 33], and considering an alpha of 0.05 a power of 80% assuming a usual correlation between them of 0.1 (Cohen, 1992) [34], we estimated that a total of 70 children who completed the study and provided usable data were required per study group to detect a difference of proportions of 10 percentage points between the WL (i.e. traditional and numeric) and comparison groups. Considering the four study groups we estimated that an overall sample size of at least 280 school children was required (70 children per study group). We estimated a rate of incomplete information of 10% for the overall recruited sample. Differences in the characteristics of the four study groups were explored by using chi-square test.

Comparisons between WL (WL and WL + C) and NF (NF and NF + C) groups were used to explore the overall objective understanding of WL and the time required to make a decision. To examine the objective understanding of WL, logistic regression models were fitted to estimate the adjusted percentage (by sex, age and school cluster) of children correctly identifying the healthiest option, the least healthy option and both, across these groups. To examine differences in the time required to make a decision, quantile regression models were fitted to estimate the adjusted median time (by sex, age and school cluster) across NF or WL groups.

Differences in the objective understanding of traditional and numeric WL, as well as in the effectiveness of communication strategies to explain the correct use of WL, were explored among children assigned to WL groups (WL and WL + C) only. To explore differences in the objective understanding between traditional and numeric WL, logistic regression models were fitted to estimate the adjusted percentage (by sex, age and school cluster) of children correctly identifying the healthiest option, the least healthy option and both, across products labeled with traditional and numeric WL. Differences in the time required to make a decision were also explored among these groups using a similar approach as the one described above.

To compare the effectiveness of communication strategies to explain the correct use of WL, the adjusted percentage of children correctly identifying the healthiest product, the least healthy product or both across communication strategies (video or poster) was estimated through logistic regression models. Generalized linear regression models adjusted by sex, age and cluster (school) were used to estimate the difference of median of seconds among communication strategies.

To estimate the impact of cartoon characters in the ability of children to choose correctly, a logistic regression model was fitted to estimate the adjusted percentage (by sex, age and school cluster) of children correctly identifying the healthiest product, the unhealthiest product and both, across cartoon character groups. Stratified proportions for NF and WL groups were displayed.

Stata v14 software was used to develop the statistical analysis.

## Results

A total of 410 elementary school children were included in this study **(**Table 1**).** Half were males, more than half were aged between 6 and 9 years old. Most participants were from School #2. Less than 10 percent of the sample reported not being able to read. No differences were observed across study groups.

**Table 1:** Socio-demographic characteristics of children

		NF groups (Control)		WL groups		***p*** value
Study group n (%)	Total 410 (100)	NF 120 (29.3)	NF + C 85 (20.0)	WL 109 (26.6)	WL + C 96 (23.4)	
	n (%)	n (%)	n (%)	n (%)	n (%)	
**Sex**						
Male	205 (50.0)	63 (62.5)	45 (52.9)	54 (49.5)	43 (44.8)	.649
Female	205 (50.0)	57 (47.5)	40 (47.1)	55 (40.5)	53 (55.3)	
**Age**						
6–9 y old	239 (58.3)	67 (55.8)	52 (61.2)	61 (56.0)	59 (61.5)	.746
10–13 y old	171 (41.7)	53 (44.2)	33 (38.8)	48 (44.0)	37 (38.5)	
**School**						
1	100 (24.4)	35 (29.2)	14 (16.5)	31 (28.4)	20 (20.8)	.294
2	240 (58.5)	63 (2.5)	55 (64.7)	60 (55.1)	62 (64.6)	
3	70 (17.1)	22 (18.3)	16 (18.8)	18 (16.5)	14 (16.6)	
**Grade**						
1–3	208 (50.7)	61 (50.8)	47 (55.3)	52 (47.7)	48 (50.0)	.770
4–6	202 (49.3)	59 (49.2)	38 (44.7)	57 (52.3)	48 (50.0)	
**Able to read**						
Yes	376 (91.7)	109 (90.8)	78 (91.8)	100 (91.7)	89 (92.7)	.970
No	34 (8.3)	11 (9.2)	7 (8.2)	9 (8.3)	7 (7.3)	

*NF* Nutrition facts table (control), *NF + C* Nutrition Facts and cartoon character, *WL* Warning labels, *WL + C* Warning labels and cartoon character. Differences across subgroups were tested using chi square tests

Supplementary Table 2 shows the objective understanding of WL across study groups, type of warning label (traditional or numeric) and food group.

### Overall objective understanding of WL

Overall, WL led to a higher percentage of children correctly choosing the healthiest option, the least healthy option, and both (the healthiest and the least healthy option) compared to NF (*p* values < 0.05) (Table 2). Similar results were observed across traditional and numeric WL and most food categories (*p* values < 0.05), however, no differences in the percentage of children correctly choosing the healthiest candy were observed between WL and NF (Table 2). Among children who were unable to read (*n* = 34), WL also led to a higher percentage of children identifying the least healthy products such as beverages (21.9% NF vs 30.9% WL, *p* < 0.05) (data not shown).

**Table 2:** Adjusted^a^ proportions of children correctly choosing the healthiest option, the least healthy option and both, across study groups

Study groups	NF^**b**^ groups (Control) (***n*** = 205)	WL^**b**^ groups (***n*** = 205)
	% (95% CI)	% (95% CI)
**Correctly choosing the healthiest product overall**	27.3 (22.2, 32.4)	**59.4 (48.1, 70.7)**
*Traditional WL*	27.0 (8.9, 45.2)	**65.0 (54.9, 75.1)**
Beverages	24.9 (10.7, 39.1)	**53.7 (45.7, 61.7)**
Breakfast cereals	14.1 (9.3, 18.8)	**66.4 (56.9, 75.8)**
Dairy beverages	40.6 (33.0, 48.1)	**77.2 (68.6, 85.9)**
*Numeric WL*	27.9 (14.2, 41.6)	**51.2 (37.1, 65.3)**
Juices in returnable packaging	32.8 (25.9, 39.6)	**55.1 (44.8, 65.3)**
Cookies	20.9 (14.0, 27.9)	**47.7 (44.5, 51.0)**
Candies	49.2 (45.6, 52.8)	45.1 (40.5, 49.7)
**Correctly choosing the unhealthiest product overall**	21.5 (18.4, 24.4)	**54.1 (32.7, 75.5)**
*Traditional WL*	16.9 (13.3, 20.5)	**52.4 (31.6, 73.1)**
Beverages	19.6 (15.7, 23.5)	**44.8 (37.6, 51.9)**
Breakfast cereals	9.1 (3.1, 15.3)	**55.8 (45.0, 66.6)**
Dairy beverages	20.1 (18.0, 22.2)	**57.0 (46.9, 67.1)**
*Numeric WL*	28.7 (26.1, 31.4)	**56.8 (35.4, 78.2)**
Juices in returnable packaging	35.6 (32.8, 38.5)	**64.3 (58.6, 70.0)**
Cookies	21.1 (19.5, 22.6)	**49.7 (35.2, 64.2)**
Candies	26.5 (15.1, 37.9)	**52.9 (40.3, 65.4)**
**Correctly choosing the healthiest and the least healthy product overall**	24.3 (20.4, 28.3)	**56.8 (40.8, 72.8)**

^a^Adjusted proportions estimated using logistic regression models adjusted by sex, age and school cluster

^**b**^Includes both NF groups (NF and NF + C) or both WL groups (WL and WL + C)

**Bolds** indicate significantly different (*p* < 0.05) to NF groups

### Overall time required to choose products

Overall, we did not find differences between NF and WL groups in the time required to identify the healthiest or the unhealthiest product. However, differences within food categories were observed (Supplementary Table 2). Children assigned to WL groups spent less time choosing the healthiest breakfast cereal (11.8 seconds, 95%CI: 10–13.7), the healthiest juice (9.7 seconds, 95%CI: 8.5, 10.9), and the least healthy breakfast cereal (18.0 seconds, 95%CI: 15.4, 20.6), in comparison to those assigned to NF groups (16.1, 12.1 and 24.1 seconds respectively, *p* < 0.05). In contrast, WL led to higher times required to choose the healthiest (10.4, 95%CI: 9.3–11.4) and the least healthy cookies (22.6, 95%CI: 20.7, 24.5) compared to NF (12.4 and 15.2 respectively, *p* < 0.05).

### Traditional versus numeric WL

Overall, the objective understanding of traditional WL was higher than numeric WL (*p* < 0.05) (Table 3). This result was consistent when choosing the healthiest product (*p* < 0.05) and the least healthy one (*p* < 0.05) (Table 3). Similarly, the median time required to identify the products was lower for traditional WL (14.6 seconds) than for numeric WL (17.8 seconds, *p* < 0.05). Supplementary Table 3 shows the adjusted proportions of children correctly choosing the healthiest and the unhealthiest products, across study groups, type of WL and food group.

**Table 3:** Percentage of children correctly choosing the healthiest option, the least healthy option and both, and time required to make these decisions, across numeric and traditional warning labels (WL)

*n* = 205	Numeric WL	Traditional WL
	% (95%CI)	% (95%CI)
Correctly choosing the healthiest product	33.6 (27.5, 40.4)	**46.1 (39.3, 53.0)**
Correctly choosing the least healthy product	31.7 (25.6, 38.4)	**38.4 (31.9, 45.3)**
Correctly choosing the healthiest and the least healthy product overall	19.0 (14.2, 25.0)	**28.7 (22.8, 35.4)**
	**Median (95% CI)**	**Median (95% CI)**
Time to choose the healthiest product (seconds)	14.2 (11.2, 18.2)	**11.0 (6.4, 15.0)**
Time to choose the least healthy product (seconds)	21.73 (17.6, 27.1)	**17.7 (13.0, 24.3)**
Time to choose the healthiest or the least healthy product (seconds)	17.8 (14.7, 22.3)	**14.6 (8.8, 19.7)**

*WL* Warning label

**Bolds** indicate significantly different (*p* < 0.05) to numeric WL

### Cartoon characters versus no cartoon characters

Among NF groups (NF and NF+C), cartoon characters led to a lower percentage of children correctly identifying the unhealthiest dairy beverage (Table 4). Among groups without cartoons (NF and WL), the percentage of correct answers were significantly higher in WL group than in NF (Supplementary Table 3). Among NF groups (i.e., products without WL), cartoon characters led to a lower percentage of children correctly identifying the unhealthiest option when considering regular size products (traditional WL) compared to no cartoons (Supplementary Table 3).

**Table 4:** Percentage of children correctly choosing the healthiest option, the least healthy option and both, across cartoon character groups

	NF groups (Control)		WL groups	
	NF (***n*** = 120)	NF + C (***n*** = 85)	WL (***n*** = 109)	WL + C (***n*** = 96)
	% (95% CI)	% (95% CI)	% (95% CI)	% (95% CI)
**Correctly choosing the healthiest product overall**	29.7 (18.0, 41.4)	24.0 (17.0, 31.1)	63.9 (49.8, 77.9)	54.3 (28.9, 79.8)
Beverages	32.1 (10.5, 53.8)	14.9 (13.5, 16.3)	62.7 (46.3, 79.0)	43.6 (32.1, 55.2)
Breakfast cereals	11.1 (4.0, 18.2)	18.4 (12.8, 24.0)	71.3 (61.4, 81.3)	60.7 (46.0, 75.5)
Dairy beverages	43.3 (33.3, 53.2)	36.9 (29.5, 44.2)	76.5 (69.9, 83.2)	78.0 (66.8, 89.3)
Juices in returnable packaging	33.6 (27.2, 40.1)	31.6 (22.6, 40.7)	58.6 (44.9, 72.2)	51.2 (42.2, 60.2)
Cookies	23.8 (18.9, 28.7)	17.1 (6.0, 28.2)	53.3 (41.2, 65.4)	41.5 (21.6, 61.3)
Candies	NA	NA	NA	NA
**Correctly choosing the unhealthiest product overall**	23.8 (19.2, 28.5)	18.1 (9.6, 26.5)	58.7 (36.4, 81.1)	**48.9 (25.6, 72.4)**
Beverages	22.1 (18.8, 25.3)	16.1 (8.7, 23.6)	45.8 (35.4, 56.1)	43.6 (38.0, 49.3)
Breakfast cereals	10.4 (5.8, 15.0)	7.5 (0.10, 16.8)	67.9 (52.0, 83.8)	**42.3 (33.8, 50.7)**
Dairy beverages	27.1 (25.8, 28.3)	**10.4 (3.0, 17.8)**	65.2 (54.5, 75.8)	**47.9 (35.8, 60.0)**
Juices in returnable packaging	33.7 (26.4, 40.9)	38.5 (36.4, 40.6)	68.4 (65.5, 71.4)	59.6 (47.9, 71.4)
Cookies	22.9 (19.4, 26.5)	18.5 (9.8, 27.2)	48.6 (35.3, 61.9)	51.1 (35.3, 66.9)
Candies	NA	NA	NA	NA
**Correctly choosing the healthiest and the unhealthiest product overall**	26.7 (18.7, 34.7)	20.9 (14.9, 27.1)	61.5 (47.9, 75.1)	51.6 (27.5, 75.6)

*N*F Nutrition Facts, *NF + C* Nutrition Facts and Cartoon character, *WL* Warning Labels, *WL + C* Warning Labels and Cartoon character. Bold numbers indicates significantly different (*p* < 0.05) to NF or WL, accordingly

Among WL groups (WL and WL+C), cartoon characters led to a lower overall percentage of children correctly identifying the unhealthiest option compared to no cartoon characters **(**Table 4**).** This same effect was observed among some products labeled with traditional WL, such as breakfast cereals and dairy beverages, but not those with numeric WL (i.e., juices in returnable packaging and cookies). No other effects of cartoon characters were observed.

### Communication strategies to explain the correct use of warning labels

Among children assigned to WL groups, the short video led to higher odds (OR = 2.23, 95% CI: 1.42–3.53, *p* < 0.05) for correctly choosing the healthiest and the least healthy option compared to the posters. Similar results were observed when choosing he healthiest option (OR = 2.41, 95% CI: 1.44–4.04, *p* < 0.05), the least healthy option (OR = 1.57, 95%CI: 1.23–1.99, *p* < 0.05), and across numeric or traditional WL (Supplementary Table 4).

## Discussion

Results of this randomized experiment indicate that among a sample of Mexican school-aged children WL led to improved identification of healthier and less healthy food products in a reduced time compared to a control condition. Further, results suggest that although traditional WL perform better than numeric WL, both were effective in helping children identify the healthiest and the least healthy product in comparison to control groups. Results also suggest that this effect may be smaller when cartoon characters are displayed on the front of the package of food products, especially unhealthy products. Finally, the video strategy was more effective than posters to communicate the interpretation of how to use the WL among scholar children.

Results of this experiment are in line with other studies suggesting that traditional WL are more effective than control conditions or other labelling formats for helping children and adolescents identify healthier food products [25, 27, 28, 33]. For example, Arrúa et al., found that among Uruguayan school-aged children WL led to a higher percentage of children correctly identifying healthy food products than multiple-traffic lights [27]. Similarly, Lima et al., found that in 9–12 y old Brazilian children WL reduced the healthfulness perception of frosted flakes compared to the GDA system [28]. Similar results have also been reported among adult populations [25, 28, 33].

This study also provided relevant information regarding the time required to making food choices. Studies among adults report that the time required to select food products is between 0.04 to 18 seconds [35–37], however scarce evidence exists regarding the time required by children to complete similar tasks. According to our results, the time required to identify healthy or unhealthy foods by using WL was shorter than 20 seconds, which is similar to the average time found in Mexican adult consumers when asked to choose the least healthy option among three food products [38]. In sum, results of this study support the fact that WL are easily and quickly understood among children attending public schools in Mexico, where some are not able to read yet. Based on the former, it seems fair to suggest that WL in traditional or numeric formats have the potential of being understood among illiterate populations, which are more frequent among low- and middle-income countries [39].

In our study, we were able to explore differences in the objective understanding of traditional WL as well as numeric WL, a new WL format considered by the Mexican regulation for small products and products in returnable packaging. To date, no other studies have explored the effect of this novel WL approach. However, in line with studies indicating that different formats of WL (i.e., a triangle in Brazil or a magnifying glass in Canada) may foster better healthy food choices [37], results of this study indicate that numeric WL have the potential to inform children when products have a higher content of critical nutrients. Considering that a high percentage of small products target young populations (e.g., candies, chocolates, snacks), and previous experiences in other countries indicate that these products are not labelled [40], it seems reasonable to consider an alternate strategy to foster healthier food choices among these sorts of products. Hence, it seems possible that labelling regulations considering numeric WL for small products may be more effective in promoting healthy food choices compared to those only considering traditional WL or other non-interpretative FOPL [27, 28]. However, the effect of any front-of-pack labelling is influenced not only by the objective understanding of the label, but by a myriad of factors which were not considered in this study [25, 26]. Future studies exploring the real-life effect of WL among Mexican populations may help address this question.

Children are a main target for food marketing, with the strongest strategy focusing on generating remarkable familiarity mostly with contextual animated cartoon characters [41]. In our experiment, cartoon characters displayed on the front of the package of food products labelled with WL led to a lower percentage of children correctly identifying the least healthy product, but not the healthiest option. Contrary to our expectations, for some food groups as the breakfast cereals and milk, products with cartoon characters were perceived as the unhealthiest items (data not shown). This finding is in concordance with other studies among adults showing that products with cartoon characters were perceived as of lower nutritional quality when compared to products without these characters [42]. Taken together, results indicate that cartoon characters influence the effect of WL underscoring the need of regulating marketing strategies directed to children on unhealthy food products along with the implementation of front-of-package labelling regulations.

The need of effective communication strategies when implementing new front of pack labelling regulations has been highlighted by international agencies [43]. However, scarce evidence exists on the most effective formats. A recent study found that emoji was useful to associate food healthfulness perception with child emotions by using WL [31]. In our study, we found that a short one-minute video was more effective than emoji posters to communicate the correct interpretation of the WL. It is possible that exposures to more detailed or different explanations could have increased effects in the objective understanding of WL, especially for younger children [44]. Therefore, the authors recommend that the implementation of WL in Mexico is accompanied by a mass media campaign to inform about the correct interpretation of WL system and the inclusion of informative videos, as has been done in other countries during the implementation of new front of package labels [11, 29, 43].

The implementation of WL in Chile showed that young children had positive attitudes towards WL and became promoters of change in their families [45]. This experience underscores the importance of ensuring the correct understanding, acceptability and use of WL in Mexican children. To our knowledge, this is the first randomized experiment testing the objective understanding of WL, including the new numeric format, among Mexican school-aged children. Strengths of this study include the use of a randomized design, ensuring that the influence of confounding from observed and unobserved factors was minimal. Also, the study considered different types and sizes of foods and beverages, increasing the statistical power of our results. Finally, this study provides key information for decision-makers in Mexico regarding the objective understanding of the Mexican WL and contributes to fill the gap in knowledge regarding objective understanding of WL in children [27, 28]. However, results of this study should be interpreted considering its limitations. First, the recruitment process was not intended to provide a representative sample of Mexican school-aged children. However, the sample approximates the demographic profile of Mexican children attending public schools, representing children from the most vulnerable populations in Mexico. Second, we conducted an experiment in a controlled situation and using mock products, limiting the ability of the study to replicate a real shopping experience. Finally, only one type of cartoon character was considered. Although this character was among the most popular during the field work period, effects could vary when using different types of cartoon characters.

## Conclusions

The results of this study carried out in Mexican children aged 6 to 13 years contribute to support the evidence in Latin America regarding the potential of WL to help young consumers identify healthy and unhealthy packaged-foods in an easy and quick manner. Importantly, numeric WL considered in the Mexican and recently in the Argentinian front-of-pack-labeling regulation, also seemed to be effective to help children identify healthy and unhealthy products, suggesting that these labels aimed at unhealthy small products may effectively communicate the excessive content of nutrients of concern among children. Results also indicate that cartoon characters displayed on the front-of-the package may reduce the objective understanding of WL, underscoring the need to regulate advertising directed to children along with the implementation of front-of-pack labeling regulations. Finally, results can be drawn on to inform the work of other researchers or stakeholders interested in designing communication campaigns aimed at improving the use and understanding of front of pack labeling formats for these population groups.

## Supplementary Information

**Additional file 1.**

**Additional file 2.**

## Data Availability

The datasets used and/or analyzed during the current study are available from the corresponding author on reasonable request.

## Abbreviations

FOPL: Front-of-the-pack-labeling
WL: Warning labels
NF: Nutrition facts
GDA: Guideline Daily Amount
WLC: Warning labels and cartoon characters
NFC: Nutrition facts and cartoon characters
