# Supplementary material for: Objective understanding of front of pack warning labels among Mexican children of public elementary schools. A randomized experiment
Source: Nutr J. 2022 Jul 22;21:47. doi: 10.1186/s12937-022-00791-z (PMC9306177; doi:10.1186/s12937-022-00791-z)
Supplement: Supplementary file 2 — Additional file 2. [file 12937_2022_791_MOESM2_ESM.docx]

**Supplementary Figure 1. Study and sample distribution diagram.**

**
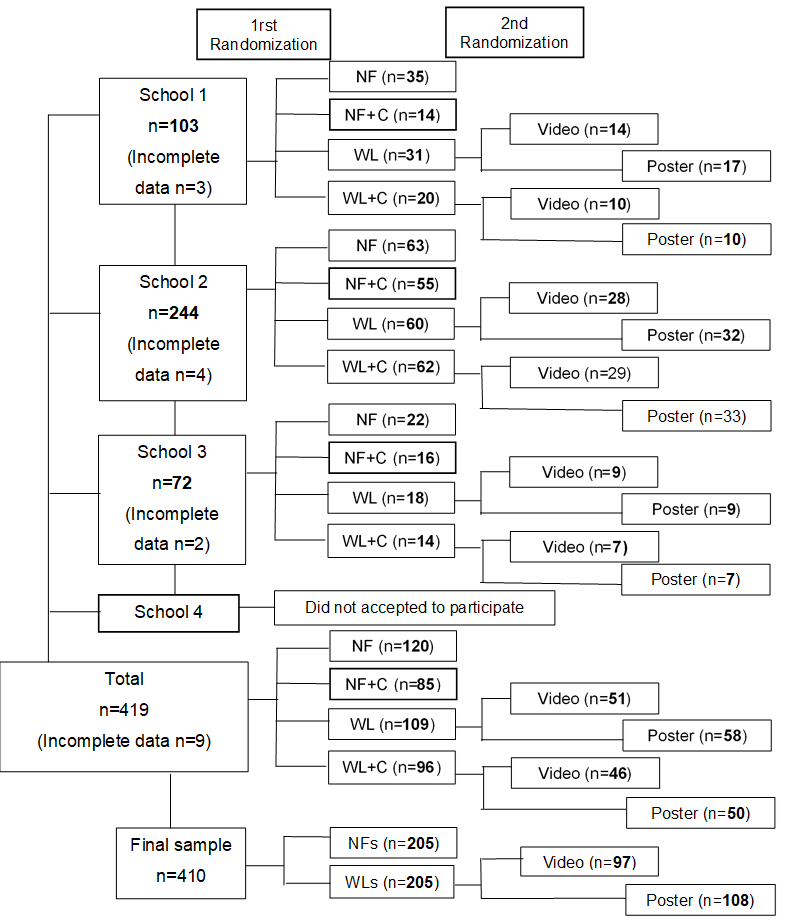
**

NF: Nutrition Facts Panel; NF+C: Nutrition Facts Panel with cartoon characters; WL: Warning Labels and WL+C: Warning Labels with cartoon characters; NFs: Nutrition Facts Panel + Nutrition Facts Panel with cartoon characters; WLs: Warning Labels + Warning Labels with cartoon characters.

**Supplementary Table 1. Mexican Nutrient profile model.**

| Eligibility | Energy | Sugar | Saturated fat | Trans | Sodium | Other thresholds |
| --- | --- | --- | --- | --- | --- | --- |
| Foods and beverages with added free sugars, fat or sodium | ≥275 kcal/100 gr; ≥70 kcal/100 ml or ≥8 kcal/100 ml from free sugars | ≥10% of total energy from free sugars | ≥10% of total energy from saturated fat | ≥1% of total energy from trans fats | ≥1 mg of sodium per 1 kcal or ≥300mg; ≥45mg for non-caloric beverages | Presence of non-nutritive sweeteners and added caffeine |
|  | 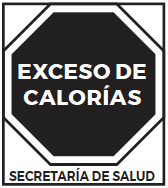 | 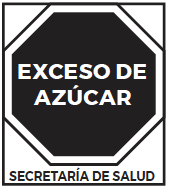 | 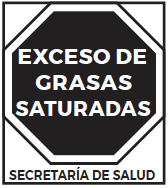 | 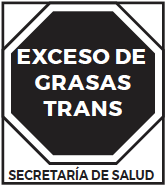 | 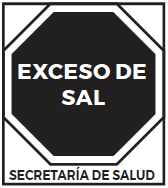 | 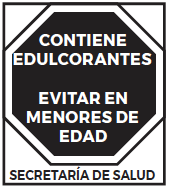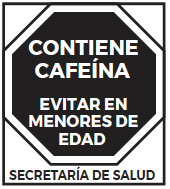 |

**Supplementary Table 2.** Adjusted^1^ median time (seconds) required to choose the healthiest and the least healthy option, by study groups and across all NF groups and all WL groups.

| **Groups of study** | **NF groups^2^**  **(n=205)** | **WL groups^2^**  **(n=205)** |
| --- | --- | --- |
|  | % (95 % CI) | % (95 % CI) |
| **Correctly choosing the healthiest and the least healthy product overall*** | 16.6 (15.2, 18.1) | 15.3 (13.9, 16.8) |
| **Correctly choosing the healthiest product overall*** | 13.5 (12.2, 14.8) | 12.2 (10.9, 13.5) |
| Beverages | 12.0 (10.4, 13.7) | 13.6 (11.9, 15.3) |
| Breakfast cereals | **16.1 (14.3, 18.9** | **11.8 (10.0, 13.7)** |
| Dairy beverages | 14.0 (12.4, 15.7) | 9.9 (8.3, 11.6) |
| Juices in returnable packaging^3^ | **12.1 (10.9, 13.4)** | **9.7 (8.5, 10.9)** |
| Cookies^3^ | **13.3 (11.8, 14.7)** | **15.4 (13.9, 16.9)** |
| Candies^3^ | 10.3 (9.2, 11.4) | 10.4 (9.3, 11.4) |
| **Correctly choosing the least healthy product overall*** | 19.6 (17.6, 21.5) | 19.3 ((17.4, 21.2) |
| Beverages | 20.8 (18.4, 23.2) | 23.5 (21.1, 25.9) |
| Breakfast cereals | **24.1 (21.5, 26.6)** | **18.0 (15.4, 20.6)** |
| Dairy beverages | 19.9 (17.7, 22.1) | 17.5 (15.3, 19.7) |
| Juices in returnable packaging^3^ | 16.9 (15.2, 18.6) | 15.8 (14.1, 17.5) |
| Cookies^3^ | **16.3 (14.4, 18.2)** | **22.6 (20.7, 24.5)** |
| Candies^3^ | 15.3 (14.0, 16.7) | 16.6 (15.3, 17.9) |

NF: Nutrition facts, WL: warning labels.

**^1^** Adjusted median times estimated using quantile regression models adjusted by sex, age and school cluster

**^2^** Figures correspond to both NF groups (NF and NF+C) or both WL groups (WL and WL+C)

^3^ Numeric WL were tested in the food group.

**Bolds** indicate significant differences between groups (p<0.05

**Supplementary Table 3.** Adjusted proportions of children correctly choosing the healthiest and the unhealthiest products, across study groups, type of warning label and food group.

|  | **Groups of study** | **NF** | **NF+C** | **Both NF groups** | **WL** | **WL+C** | **Both WL groups** |
| --- | --- | --- | --- | --- | --- | --- | --- |
|  | **n sample** | 120 | 85 | 205 | 109 | 96 | 205 |
|  | | % (95 % CI) | % (95 % CI) | % (95 % CI) | % (95 % CI) | % (95 % CI) | % (95 % CI) |
| **Correct answers overall** | | **26.7 (18.7, 34.7)** | **20.9 (14.9, 27.1)** | **24.3 (20.4, 28.3)** | **61.5 (47.9, 75.1)^ab^** | **51.6 (27.5, 75.6)^b^** | **56.8 (40.8, 72.8)^£^** |
|  | |  |  |  |  |  |  |
| **Correctly choosing the healthiest product overall*** | | **29.7 (18, 41.4)** | **24.0 (17, 31.1)** | **27.3 (22.2, 32.4)** | **63.9 (49.8, 77.9)^ab^** | **54.3 (28.9, 79.8)^b^** | **59.4 (48.1, 70.7)^£^** |
| Traditional WL | Beverages | 32.1 (10.5, 53.8) | 14.9 (13.5, 16.3) | 24.9 (10.7, 39.1) | 62.7 (46.3, 79)^ab^ | 43.6 (32.1, 55.2)^b^ | 53.7 (45.7, 61.7)^£^ |
|  | Breakfast cereals | 11.1 (4, 18.2) | 18.4 (12.8, 24) | 14.1 (9.3, 18.8) | 71.3 (61.4, 81.3)^ab^ | 60.7 (46, 75.5)^ab^ | 66.4 (56.9, 75.8)^£^ |
|  | Dairy beverages | 43.3 (33.3, 53.2) | 36.9 (29.5, 44.2) | 40.6 (33.0, 48.1) | 76.5 (69.9, 83.2)^ab^ | 78.0 (66.8, 89.3)^ab^ | 77.2 (68.6, 85.9)^£^ |
|  | Average | 29.4 (3.5, 55.3) | 23.8 (15.1, 32.6) | 27.0 (8.9, 45.2) | 69.4 (56.6, 82.2)^ab^ | 60.0 (37.0, 83.0)^b^ | 65.0 (54.9, 75.1)^£^ |
| Numeric WL | Juices | 33.6 (27.2, 40.1) | 31.6 (22.6, 40.7) | 32.8 (25.9, 39.6) | 58.6 (44.9, 72.2)^ab^ | 51.2 (42.2, 60.2)^ab^ | 55.1 (44.8, 65.3)^£^ |
|  | Cookies | 23.8 (18.9, 28.7) | 17.1 (6.0, 28.2) | 20.9 (14.0, 27.9) | 53.3 (41.2, 65.4)^ab^ | 41.5 (21.6, 61.3)^ab^ | 47.7 (44.5, 51.0)^£^ |
|  | Candies | NA | NA | 49.2 (45.6, 52.8) | NA | NA | 45.1 (40.5, 49.7) |
|  | Average | 29.8 (17.4, 42.2) | 25.3 (7.2, 43.4) | 27.9 (14.2, 41.6) | 55.5 (39.4, 71.6)^ab^ | 46.4 (15.4, 77.3)^b^ | 51.2 (37.1, 65.3)^£^ |
|  |  |  |  |  |  |  |  |
| **Correctly choosing the unhealthiest product overall*** | | **23.8 (19.2, 28.5)** | **18.1 (9.6, 26.5)** | **21.5 (18.4, 24.4)** | **58.7 (36.4, 81.1)^ab^** | **48.9 (25.6, 72.4)^bc^** | **54.1 (32.7, 75.5)^£^** |
| Traditional WL | Beverages | 22.1 (18.8, 25.3) | 16.1 (8.7, 23.6) | 19.6 (15.7, 23.5) | 45.8 (35.4, 56.1)^ab^ | 43.6 (38, 49.3)^ab^ | 44.8 (37.6, 51.9)^£^ |
|  | Breakfast cereals | 10.4 (5.8, 15) | 7.5 (0.1, 16.8) | 9.1 (3.1, 15.3) | 67.9 (52, 83.8)^ab^ | 42.3 (33.8, 50.7)^abc^ | 55.8 (45.0, 66.6)^£^ |
|  | Dairy beverages | 27.1 (25.8, 28.3) | 10.4 (3, 17.8)^a^ | 20.1 (18.0, 22.2) | 65.2 (54.5, 75.8)^ab^ | 47.9 (35.8, 60)^abc^ | 57.0 (46.9, 67.1)^£^ |
|  | Average | 20.6 (19.6, 21.5) | 11.6 (4.2, 19.1)^a^ | 16.9 (13.3, 20.5) | 59.2 (33.0, 85.4)^ab^ | 44.8 (25.4, 64.2)^abc^ | 52.4 (31.6, 73.1)^£^ |
| Numeric WL | Juices | 33.7 (26.4, 40.9) | 38.5 (36.4, 40.6) | 35.6 (32.8, 38.5) | 68.4 (65.5, 71.4)^ab^ | 59.6 (47.9, 71.4)^ab^ | 64.3 (58.6, 70.0)^£^ |
|  | Cookies | 22.9 (19.4, 26.5) | 18.5 (9.8, 27.2) | 21.1 (19.5, 22.6) | 48.6 (35.3, 61.9)^ab^ | 51.1 (35.3, 66.9)^ab^ | 49.7 (35.2, 64.2)^£^ |
|  | Candies | NA | NA | 26.5 (15.1, 37.9) | NA | NA | 52.9 (40.3, 65.4)^£^ |
|  | Average | 28.8 (17.5, 40) | 28.8 (16.4, 41.2) | 28.7 (26.1, 31.4) | 58.3 (43.5, 73.1)^ab^ | 55.2 (25.5, 85)^b^ | 56.8 (35.4, 78.2)^£^ |
| NF: Nutrition facts (comparison), NF+C: Nutrition Facts and cartoon character, WL: warning labels, WL+C: warning labels and cartoon character.  All NF groups: includes NF and NF+C groups. All WL groups: includes WL and WL+C groups  Significantly different (p<0.05) to ^a^NF, ^b^NF+C, ^c^WL, ^£^all NF groups, ^§^to traditional WL. Estimations obtained from logistic and linear regression models adjusted by age, sex and school (cluster). | | | | | | | |

**Supplementary Table 4.-** Odds for correctly choosing the healthiest or the least healthy option among children assigned to warning label groups, by communication strategy (n=205).

| **Ref. (Poster)** | O.R. (95 % CI)^1^ |
| --- | --- |
| **Correctly choosing the healthiest or the least healthy product overall** | **2.23 (1.42, 3.53)** |
|  |  |
| **Correctly choosing the healthiest product overall** | **2.41 (1.44, 4.04)** |
| Traditional WL | **2.31 (1.56, 3.43)** |
| Numeric WL | **1.62 (1.05, 2.51)** |
|  |  |
| **Correctly choosing the unhealthiest product overall** | **1.57 (1.23, 1.99)** |
| Traditional WL | **1.27 (1.09, 1.49)** |
| Numeric WL | **1.59 (1.09, 1.7**9) |

^1^Odds ratios estimated with a generalized linear models adjusted by sex, age and cluster (school).

Bolds indicate significant odds (P-value <0.05)

Image group (n=108), Video group (n=97)
